# Compound Prioritization through Meta-Analysis Enhances the Discovery of Antimicrobial Hits against Bacterial Pathogens

**DOI:** 10.3390/antibiotics10091065

**Published:** 2021-09-02

**Authors:** Loic Deblais, Gireesh Rajashekara

**Affiliations:** Food Animal Health Research Program, Department of Veterinary Preventive Medicine, The Ohio State University, OARDC, Wooster, OH 44691, USA; deblais.1@osu.edu

**Keywords:** small molecules, virtual screening, high throughput screening, phytopathogens, animal pathogens, foodborne human pathogens

## Abstract

The development of informatic tools to improve the identification of novel antimicrobials would significantly reduce the cost and time of drug discovery. We previously screened several plant (*Xanthomonas* sp., *Clavibacter* sp., *Acidovorax* sp., and *Erwinia* sp.), animal (Avian pathogenic *Escherichia* *coli* and *Mycoplasma* sp.), and human (*Salmonella* sp. and *Campylobacter* sp.) pathogens against a pre-selected small molecule library (*n* = 4182 SM) to identify novel SM (hits) that completely inhibited the bacterial growth or attenuated at least 75% of the virulence (quorum sensing or biofilm). Our meta-analysis of the primary screens (*n* = 11) using the pre-selected library (approx. 10.2 ± 9.3% hit rate per screen) demonstrated that the antimicrobial activity and spectrum of activity, and type of inhibition (growth versus virulence inhibitors) correlated with several physico-chemical properties (PCP; e.g., molecular weight, molar refraction, Zagreb group indexes, Kiers shape, lipophilicity, and hydrogen bond donors and acceptors). Based on these correlations, we build an in silico model that accurately classified 80.8% of the hits (*n* = 1676/2073). Therefore, the pre-selected SM library of 4182 SM was narrowed down to 1676 active SM with predictable PCP. Further, 926 hits affected only one species and 1254 hits were active against specific type of pathogens; however, no correlation was detected between PCP and the type of pathogen (29%, 34%, and 46% were specific for animal, human foodborne and plant pathogens, respectively). In conclusion, our in silico model allowed rational identification of SM with potential antimicrobial activity against bacterial pathogens. Therefore, the model developed in this study may facilitate future drug discovery efforts by accelerating the identification of uncharacterized antimicrobial molecules and predict their spectrum of activity.

## 1. Introduction

Technological advances in synthetic chemistry have made available billions of novel molecules with uncharacterized antimicrobial properties [1]; however, the development of novel antimicrobial agents can be expensive (over US $1 billion) and time consuming (12–15 years) [2]. This is especially true with the use of large random small molecule (SM) libraries where extensive high-throughput screenings are required to identify SM candidates with desired properties (hit compounds). Further, random SM libraries are often associated with low success rate (identification of novel hit compounds below 0.5%), which makes drug discovery an unattractive sector for industrial development [2]. Thereby, there is a crucial need to identify interconnections between the properties of a molecule and its phenotype that will aid developing informatic tools minimizing the drug discovery efforts and cost while improving the identification and discovery of novel therapeutics in order to mitigate the antimicrobial resistance burden [3,4,5]. Virtual in silico screening is a useful approach to minimize the work required for the identification and development of lead antimicrobial compounds [6]. For example, it allows identifying SM with promising druggable properties or design in silico small tailored libraries composed of SM likely to be active against a desired biological target. However, the in silico prediction and design of SM with desired biological properties rely on the outputs obtained from in vitro screenings. Therefore, it is essential to understand the relationships between the SM chemical structure and its antimicrobial activity.

The study of chemical structures within compounds of interest has been for a long time a key criterion for the classification of antibiotics [7]. To date, several functional groups have been identified with specific antimicrobial effects [7,8]. The antimicrobial agents can be classified into groups harboring distinct chemical structures (e.g., β-lactams, aminoglycoside, macrolides, quinolones and fluoroquinolones, streptogramins, sulfonamides, tetracyclines, and nitroimidazoles) [7,8]. These characteristic chemical structures are associated with specific antimicrobial properties (e.g., narrow versus broad-spectrum; anti-viral/bacterial/fungal/parasitic), toxicity, and mode of actions (e.g., inhibitors of cell wall, nucleic acids or protein synthesis, or membrane function) [7,8]. However, the antimicrobial activity of a designated molecule may also be influenced by other parameters, such as the presence of radicals surrounding the functional group, the concentration of molecule tested, and the physico-chemical properties (PCP) of molecules. In fact, several studies highlighted the associations between the PCP of molecules and the characterization of specific phenotypes (i.e., antimicrobial activity and anti-oxidant/cancer properties) [9,10,11]. The Lipinski rule of five is a standard for the discovery of potential therapeutic molecules with druggable properties [12,13]. A previous study developed an in silico model facilitating the identification of hit molecules with antimicrobial activity against several model organisms (i.e., yeast, bacteria, and nematodes) [14]. More precisely, a pre-selected library of 7500 SM was built from an initial library of 81,000 uncharacterized synthetic SM using a three-way approach: 1) enrichment of library in vitro using *Saccharomyces cerevisiae* as the model; 2) prioritize the compounds using two-property filter (lipophilicity (LogP) and Lipinski hydrogen acceptors (HBA)); and 3) predict the phenotypes of the selected molecules using a naïve Bayes model. The pre-selected library showed an increased rate in the identification of SM with growth inhibition properties in vitro (up to 16-fold) against several model organisms (e.g., *Escherichia coli*, *Candida albicans*, *Caenorhabditis elegans*, and *Bacillus subtilis*) compared to the original library. From this subset, in our earlier studies, we screened a library of 4182 compounds to identify compounds effective against several plant (*Xanthomonas*, *Erwinia*, *Clavibacter*, and *Acidovorax*), animal (*Mycoplasma* and *Escherichia*), and foodborne-human (*Salmonella* and *Campylobacter*) pathogens that are of public health and economic importance [15,16,17,18,19,20,21,22,23,24,25,26]. The objective of this study was to characterize and associate the antimicrobial properties of the pre-selected library based on their PCP. Our studies provide novel insights for the prediction of uncharacterized molecules with potential antimicrobial activity and enable enriching the random libraries with SMs that are more likely to have antimicrobial activity, thus accelerating drug discovery efforts.

## 2. Results

### 2.1. Compounds’ Prioritization Increases the Identification of Antimicrobials Effective against Bacterial Pathogens with Diverse Taxonomic and Host Range Profile

For in silico analyses, we used the in vitro or in vivo data obtained from our earlier published studies using high throughput screening of 4182 compounds on nine different pathogens [15,16,17,18,19,20,21,22,23,24,25,26]. This library was screened at the specified concentration (between 10 and 200 µM) against each pathogen (Table 1). The data associated with (1) the type of inhibition (growth versus virulence) were recorded to determine whether growth inhibitors possess different PCP profiles compared to virulence inhibitors, and (2) the spectrum of the activity of the SM was recorded to determine whether narrow- and broad-spectrum hits possess characteristic PCP profiles. Further, the lead compounds were separated from the hit compounds to determine whether lead compounds possess a different PCP profile compared to hit compounds. Additional details about the experiments performed to select and validate the antimicrobial potency of these compounds (i.e., dose-response assays, activity spectrum assays on beneficial and other pathogenic bacteria, antimicrobial efficacy in planta and in chickens, microbiome studies) are described in our published studies [15,16,17,18,19,20,21,22,23,24,25,26]. By analyzing the previous eleven primary screening data, 2073 SM (49.6%) were identified as hits (SM that completely inhibited the growth or attenuated at least 75% virulence of the designated pathogen(s); Supplementary File 1). A hit rate (number of growth/virulence inhibitors hit compounds obtained out of the 4182 SM tested) of 10.2% ± 9.3% per screen was observed across the nine bacterial species (Table 1). The hit rate of the screenings was not associated with the taxonomic diversity of the species studied. However, avian pathogenic *E. coli* (APEC), *Salmonella enterica* subsp. *enterica* serotype Typhimurium (ST), and *Acidovorax citrulli* (*Ac*) harbored a lower hit rate (between 0.5% and 1.4%) compared to the other screenings (>11.1%) despite the use of limited nutrient growing conditions (Table 1). Interestingly, the hit rate for virulence inhibitor screenings was high as well (2.4% for APEC quorum sensing and 5.2% for ST biofilm inhibitors; Table 1), and at least 50.6% of them were identified as specific virulence inhibitors with no growth inhibiting effect on the other species tested (Supplemental File 1). The type of inhibition (growth versus virulence) was recorded (Supplemental File 1) to determine whether growth inhibitors harbor different PCP profiles compared to virulence inhibitors.

A total of 1147 hits harbored antimicrobial activity against more than one bacterial species tested (hits affecting between two to eight species; Table 2) and 1254 hits were active against specific pathogens categories; 29% (*n* = 199/691) were specific to animal pathogens (*n* = 2), 34% (*n* = 327/951) were specific of human foodborne pathogens (*n* = 2), and 46% (*n* = 728/1581) were specific to phytopathogens (*n* = 5; Table 2). Forty-five percent of the hits (*n* = 926) were active against only one bacterial pathogen tested (*n* = 26 growth inhibitors for *Erwinia tracheiphila* (*Et*), *n* = 63 growth inhibitors for *Clavibacter michiganensis michiganensis* (*Cmm*); *n* = 1 growth inhibitor for *Ac*; *n* = 56 and 346 growth inhibitors for *Xanthomonas gardneri* (*Xg*) and *perforans* (*Xp*); *n* = 127 growth inhibitors for *Mycoplasma gallisepticum* (*Mg*); *n* = 136 growth inhibitors for *Campylobacter jejuni* (*Cj*); *n* = 55 quorum sensing inhibitors for APEC; *n* = 1 growth and 115 biofilm inhibitors for ST. The spectrum of activity of the SM was recorded (Supplemental File 1) to determine whether narrow- and broad-spectrum hits possess characteristic PCP profiles.

A total of 18 lead compounds were identified from our previously published drug discovery studies (Table 3 and Supplemental File 1) [15,16,17,18,19,20,21,22,23,24,25,26]. These compounds have been shown to significantly reduce the load of designated pathogens and severity of the disease in vivo, with minimal impact on host, its microbiota, commensal bacteria, and probiotics/biocontrols. In addition, 73 compounds with promising antimicrobial properties for future development as lead compounds were identified across the eleven primary screens (Supplemental File 1). These compounds have been shown to significantly reduce the load of the designated pathogens in vitro, with minimal impact on commensal bacteria and probiotics/biocontrols, but with limited antimicrobial efficacy in vivo. Additional details about the experiments performed to select and validate the antimicrobial potency of these compounds (*n* = 18 + 73) are described in our published studies [15,16,17,18,19,20,21,22,23,24,25,26]. These 91 compounds were used (Supplemental File 1) to determine whether lead compounds possess different PCP profile compared to hit compounds. 

### 2.2. The Antimicrobial Activity and Spectrum of Activity of the SM Correlated with Specific Physico-Chemical Properties

**Figure 1:**
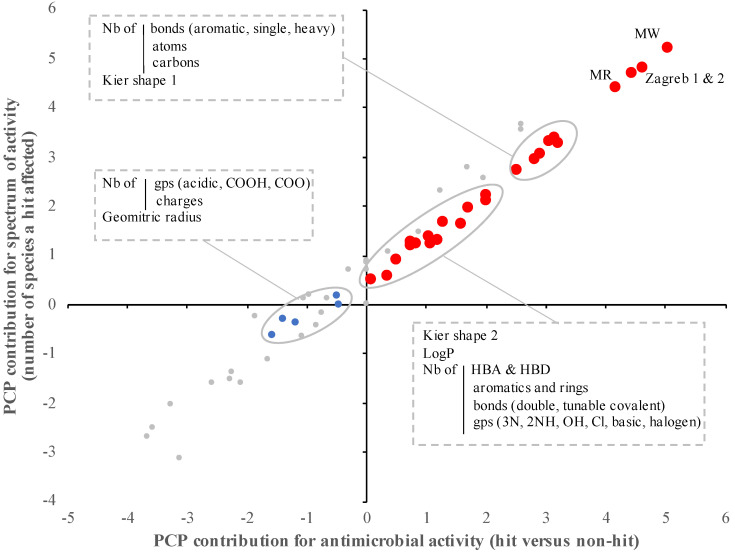
Impact of physico-chemical properties (PCP) on the antimicrobial activity and spectrum of activity of the small molecules (SM). The antimicrobial activity/activity spectrum model was built based on the in vitro data from the primary screenings (*n* = 11 screenings) and the PCP of the SM obtained from multiple reference database (ChemBridge, PubChem, Joelib, ChemMine, and OpenBabel). The contribution of each PCP was determined based on the bootstrap forest method. The contribution score was log-transformed. Red dots represent PCP significantly associated with both antimicrobial activity and spectrum of activity of the SM (*p* < 0.01). Blue dots represent PCP significantly associated with only the spectrum of activity of the SM (*p* < 0.01). Gray dots represent PCP not associated with antimicrobial activity and spectrum of activity of the SM (*p* > 0.001). Nb: number; Gp: group; LogP: lipophilicity; HBA: hydrogen bond acceptor; HBD: hydrogen bond donor.

Among the 60 PCP studied, 24 PCP were significantly associated with antimicrobial activity (hits versus non-hits) of the SM (*p* < 0.01; Figure 1 and Table 4). More precisely, the hits identified across the nine bacterial pathogens tested (*n* = 2073) were characterized by significantly higher molecular weight (MW), lipophilicity (LogP), Kier shape, the number of bonds (aromatic, heavy, hydrogen bond donor (HBD)), number of groups (aromatic, atom, amine, basic, chlorine, carbon, halogen, hydroxy, and ring), Zagreb group index, and molar refractivity (MR), but significantly lower number of azide group, hydrogen bond donor acceptor (HBA) and double bonds compared to the SM with no antimicrobial activity (*p* < 0.01; Figure 1). It is important to mention that 50% of the PCP described above (*n* = 12/24, especially MW, MR, and Zagreb indexes) were predicted to have a higher contribution to the antimicrobial activity than the other half (contribution score up to 10,000-fold different between the two populations; Table 4). Similar trends were observed when the PCP was compared with the spectrum of activity (number of species affected by a designated hit) of the SM (*p* < 0.01; Figure 1 and Table 4). Overall, the contribution of the selected PCP was equivalent between the antimicrobial activity and the spectrum of activity (r^2^ = 0.97; *p* > 0.001; Figure 1).

Several broad-spectrum hits (*n* = 32; SM effective against at least seven species) were identified across the 11 screenings performed. These hits were characterized by a lower number of hydrogen bond acceptors 2 (HBA-2 from OpenBabel; *n* = 3 ± 1 HBA per SM) compared to the other hits (SM effective against less than seven species; *n* = 4.6 ± 1.3 HBA per SM) and the inactive SM (*n* = 4.4 ± 1.2 HBA per SM; Table 4). However, it is important to mention that population size of the broad-spectrum hits is small, thereby reducing the statistical power of our analysis. Similarly, the lead compounds (*n* = 18) were characterized with a significantly higher number of charges and a significantly lower number of rotatable bonds compared to the other hits (*n* = 2055; *p* < 0.01). However, these two PCP were identified with a lower contribution score (*n* < 1), perhaps because of the small population of lead compounds (Table 4). We analyzed by combining the lead compounds with the hits that displayed antimicrobial properties having potential for developing into lead compounds (*n* = 18 + 73) to increase the statistical power. These compounds were characterized by a significantly higher number of charges, nitrile, amine, and nitro groups, and a significantly lower number of rings and heterocycles compared to the other hits (*n* = 1982; *p* < 0.01; Table 4); however, only the number of charges, amine groups, heterocycles, and rings had a contribution score value above one.

A total of 23 PCP showed significant differences between the growth inhibitors’ hits (*n* = 1898) and the virulence inhibitors’ hits (*n* = 310; Table 4). The majority of the PCP (*n* = 15/23) had a contribution score above 100. Growth inhibitors were characterized by significantly higher MW, MR, Kier shapes, Zagreb indexes, lipophilicity, geometric diameter, and the number of bonds (single, heavy, HBA, and aromatic), atoms (nitrogen, hydrogen and carbon) and groups (heterocycles, basics, aromatics, and rings) compared to the virulence inhibitors (*p* < 0.01). Interestingly, only the number of double bonds was significantly lower with growth inhibitors compared to the virulence inhibitors (*p* < 0.01).

The results presented in Figure 1 identified 37 PCP significantly associated with the antimicrobial and spectrum activity of an SM (Table 2). Using these PCP, we built an in silico model that accurately classified 80.8% of SM as hit compounds from 2073 active compounds (*p* < 0.01; Supplemental File 1). Similar accuracy (81 ± 3%) was obtained when subsets were randomly selected by omitting 4.2–9.5% of our pre-selected library (*n* = 3785, 3819, and 4007 compounds per subset). Similarly, an accuracy of 81.6% ± 2.1% was obtained when three subsets of 10% randomly excluded SM library were analyzed using our in silico model. The SM identified as hits in vitro but not in silico (mis-predicted hits; *n* = 397) displayed distinct PCP profiles compared to the SM identified as hits in vitro and in silico (accurately predicted hits; *n* = 1676). A total of 24 PCP displayed significant differences. Interestingly, 21 of the 24 PCP showing significant differences between accurately predicted and mis-predicted hits also displayed significant differences between hit and nonactive SMs (all PCP except number of rings and both Zagreb groups indexes; Table 4). Out of the three PCP (geometric radius, the number of charges, and ether groups) showing only significant differences between accurately and mis-predicted hits, only the number of ether groups was significantly lower in mis-predicted hits compared to the accurately predicted hits (*p* < 0.01) and with a contribution score above one (*n* = 7.7). The mis-predicted hits were evenly distributed across the eleven screenings performed. Further, only one SM (PubChem ID 1529361, a quorum sensing inhibitor for APEC) out of 18 lead compounds (compounds that significantly reduced the pathogen load and severity in vivo) and 9% (*n* = 7/73) of the promising hits (compounds that significantly reduced the pathogen load and severity in vitro but limited impact in vivo) were mis-predicted based on our model (Supplemental File 1).

**Figure 2:**
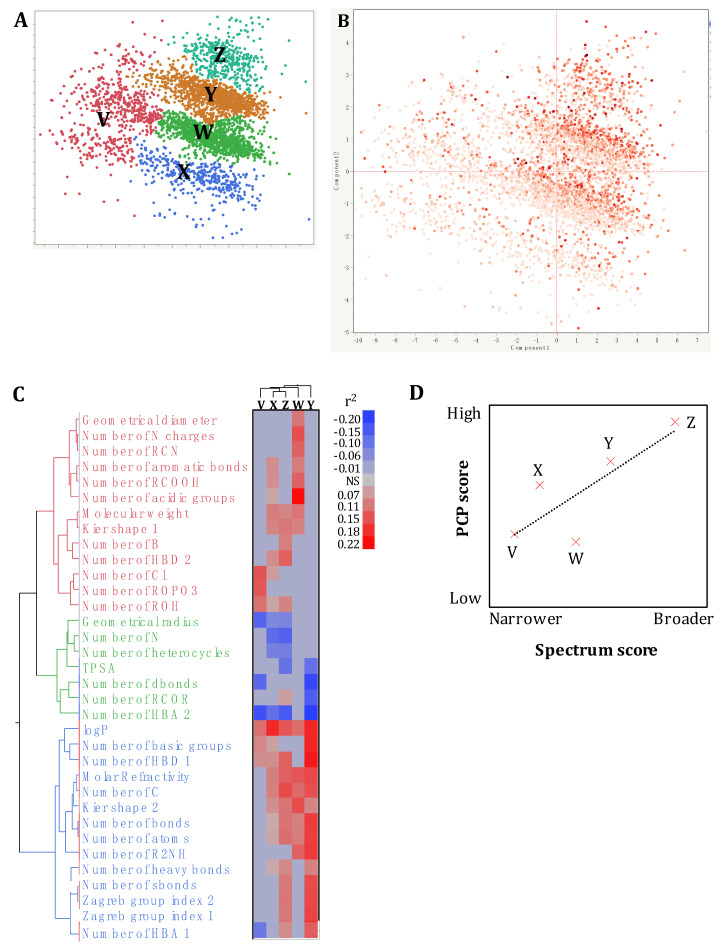
Impact of the physico-chemical properties (PCP) of small molecules (SM) on their antimicrobial activity and spectrum of activity. (**A**,**B**) Spatial distribution of the pre-selected SM library (*n* = 4182) based on their PCP using a principal component analysis plot. Component 1 and 2 explained 26.8% and 9.25% of the variability across the 4182 SM based on the 60 PCP obtained from multiple reference databases (ChemBridge, PubChem, Joelib, ChemMine, and OpenBabel). Each dot represents the distribution of a SM based on its PCP. (**A**) displays the clusterization of the SM library into 5 distinct clusters based on the PCP of the SM. (**B**) displays the spectrum of activity of each SM (dot) based on the published primary screening data. The color of the dots is proportional the spectrum of antimicrobial activity of a given SM based on the primary screenings performed (number of species affected by the SM; from 0 to 8 species). Cluster V (*n* = 594 SM), W (*n* = 1396 SM), X (*n* = 404 SM), Y (*n* = 1355 SM), and Z (*n* = 433 SM). (**C**) The two-way clustering plot was built based on the 34 PCP showing significant correlations with the spectrum of antimicrobial activity of the SM. Five clusters were generated based on the PCP profile similarities described in Figure 2A. HBA and HBD: hydrogen bond acceptor and donor, respectively. TPSA: topological polar surface area. LogP: lipophilicity. NS: nonsignificant correlation (*p* > 0.01). (**D**) Linear regression between the spectrum of activity and the PCP of the clusters (r² = 0.66). PCP score: score generated based on the number of significant (*p* < 0.01) correlation between the spectrum of activity of the SM and their PCP (Figure 2C). Spectrum score: score generated based on the hit compounds and the number of bacteria affected by hit compounds (Supplemental File 3).

A discriminant analysis combined with a principal component scoring matrix showed that the pre-selected SM library (*n* = 4182) was composed of five distinct clusters (Cluster V (*n* = 594 SM), W (*n* = 1396 SM), X (*n* = 404 SM), Y (*n* = 1355 SM), and Z (*n* = 433 SM); Figure 2A) based on the PCP studied (*n* = 60). Therefore, more than one PCP profile might to be associated with the antimicrobial activity and spectrum of activity of the SM. Cluster Z harbored highest hit rate (67.5%), followed by the cluster Y (52.1%), W (47.2%), X (35.8), and V (35%; Figure 2A). 

Interestingly, a unidirectional gradient in the spectrum of activity was observed within and between clusters (Figure 2B), suggesting that small PCP variations within each cluster have detrimental impact on the spectrum of activity of the SM. The multivariate analysis identified 28 PCP positively and seven PCP negatively correlated with the spectrum of activity of the SM across the five clusters (0.07 < r^2^ < 0.23; −0.20 < r^2^ <−0.07; *p* < 0.01; Figure 2C). Only LogP was positively correlated with the five clusters (r^2^ > 0.12; *p* < 0.01). Overall, the clusters V, X, and Z displayed different spectrum/PCP correlation profile compared to cluster W and Y (Figure 2C). The clusters Z and Y were the only clusters positively correlated with the number of heavy and single bonds and the Zagreb indexes (r^2^ > 0.09; *p* < 0.01), and negatively correlated with topological surface polar area (TPSA; r^2^ < −0.10; *p* < 0.01; Figure 2C). Interestingly, clusters Z and Y harbored the highest number of multitarget hits (Supplemental File 3), and the highest number of correlations between the PCP and the spectrum of activity (*n* = 23 and 18, respectively). Therefore, the spectrum of activity of the SM might be explained at some level by these specific PCP profiles and the number of correlations between the PCP and the spectrum of activity (linear regression r^2^ = 0.66; *p* = 0.09; Figure 2D). However, it is important to mention that only five clusters were used to test this hypothesis, which limits the statistical power of our analysis. In contrast, cluster Y had the highest correlation values (r^2^ = 0.17 ± 0.02) while cluster Z had the lowest correlation values (absolute r^2^ = 0.11 ± 0.02; Figure 2C), which suggest that the intensity (r^2^ value) of the correlations between the PCP and the spectrum of activity was not associated with the spectrum of activity of the SM (r^2^ = 0.001). 

### 2.3. Using Virtual Screening Tools to Prioritize the Selection of SM with Potential Antimicrobial Activity

**Figure 3:**
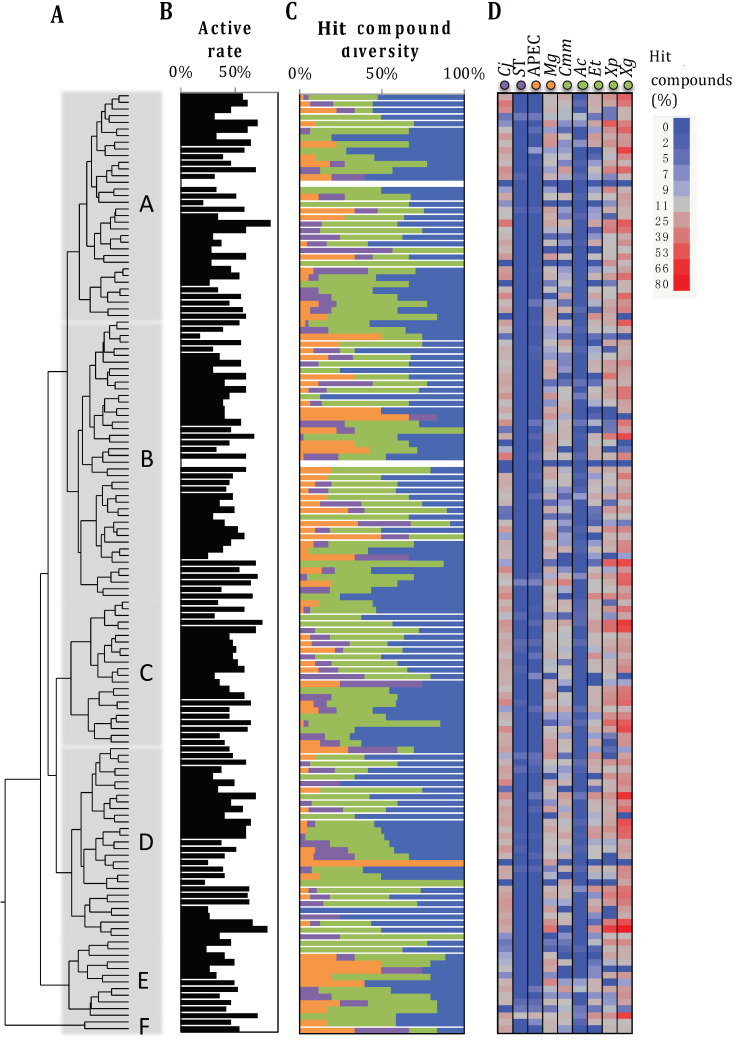
Analyses of the pre-selected small molecule (SM) library based on the two-dimension Tanimoto scoring system. (**A**) Chemical structure diversity of the SM library based on two-dimension Tanimoto scoring (PubChem database) combined with a hierarchical clustering method. The SM (4182 SM) were grouped into 141 clusters that contain between 4 and 144 SM with high chemical structure similarities (Bartlett’s test; *p* < 0.01). Cluster A: 188 SM; B: 252 SM; C: 727 SM; D: 802 SM; E: 231 SM; F: 43 SM. (**B**) Proportion of hit compounds within each cluster. (**C**) Activity spectrum of the hits within each cluster. Green, orange, and purple bars: hits specific to plant, animal, or human/foodborne pathogens, respectively at a given SM concentration and growing condition (Table 1); blue bar: hits effective against more than one pathogen category. (**D**) Proportion of the hits per pathogen (*n* = 9). The cell color (from blue to red) is proportional to the number of hits for the designated pathogen for a given SM cluster. Cj: *Campylobacter jejuni* 81–176; ST: *Salmonella enterica* subsp. *Enterica* serotype Typhimurium JSG626; APEC: avian pathogenic *Escherichia coli* O78; Mg: *Mycoplasma gallisepticum* MG37; Cmm: *Clavibacter michiganensis* subsp. *Michiganensis* C290; Ac: *Acidovorax citrulli* Xu9-15; Et: *Erwinia tracheiphila* TedCu10; Xg: *Xanthomonas gardneri* SM761; and Xp: *Xanthomonas perforans* SM755-12. Data presented in Figure (**B**–**D**) are based on the in vitro data obtained from the primary screenings (*n* = 11 screenings) of our previous studies.

The two-dimension Tanimoto scoring system (measures the molecular similarity) clustered the 4182 SM into six major groups (A–F). These groups were subdivided into total of 141 clusters (*n* = 4 to 144 SM per cluster) with highly similar chemical structures (*p* < 0.01; Figure 3). Each cluster harbored an equivalent hit rate (47.7 ± 13.8% per cluster) with equivalent spectrum of activity. Only seven clusters (*n* = 40 SM total) were composed of hits effective against a specific type of pathogen and only two clusters (*n* = 10 SM) harbored no hits (Figure 3). Thereby, the Tanimoto scoring system was inconclusive in characterizing the antimicrobial activity of the SM. Similarly, the pre-selected SM library (*n* = 4182) used in this study is composed of 92.6% of SM following the Lipinski rule of five (*n* = 3873); by consequence, the Lipinski rule of five was also inconclusive in characterizing the antimicrobial activity of the SM.

## 3. Discussion

The pre-selected SM library used in this study was created based on the empirical in silico model developed by Wallace et al. [14]. This model was previously shown to improve the identification of uncharacterized molecules with potential antimicrobial properties against several model organisms (e.g., *E. coli*, *C. albicans*, *C. elegans*, and *B. subtilis*) [14]. Our pre-selected SM library, obtained based on Wallace et al. [14], provided a high hit rate (average of 20-fold higher compared to conventional libraries [2]) despite the taxonomic diversity and host range of the bacterial pathogens screened. Therefore, our study supports the following statement from Wallace et al.: “Compounds that inhibit yeast growth are more likely to induce phenotypes in other model organisms.” [14]. It is also important to notice that nutrient availability was a decisive factor influencing the hit rate [16]. However, certain pathogens (e.g., ST, APEC, and *Ac*) were less susceptible to the pre-selected SM library (hit rate between 0.5% and 5.2%), even when limiting nutrient conditions and higher SM concentrations (200 μM instead of 100 μM) were used for the primary screens. Therefore, we hypothesize that these species might have antimicrobial resistance mechanisms (i.e., efflux pump or periplasmic enzymes) that increase their resistance to the SM [27,28]. Overall, the screening of the plant (*n* = 5), animal (*n* = 2), and foodborne-human (*n* = 2) pathogens against the pre-selected SM library allowed the identification of 18 lead compounds (SM that successfully mitigate the designated pathogen in vivo/in planta, with minimal impact on the host and its microbiota) and 73 hits with promising antimicrobial properties in vitro for the development of future lead compounds [15,16,17,18,19,20,21,22,23,24,25,26] (Supplemental File 1).

The taxonomy and host range of the screened pathogens, and the growing conditions used for the SM screens were not associated with the PCP profile of the hit compounds. On the other hand, the antimicrobial activity and the activity spectrum of a SM was associated with its PCP [29,30]. The pre-selected SM library was composed of five distinct PCP profiles with different hit compound discovery rates (between 35% and 67.5%) and the number of narrow- and broad-spectrum SM. Our empirical model accurately predicted 80.8% of the SM with antimicrobial activity (*n* = 1676/2073) based on the 11 screens performed on nine species and the 60 PCP recorded. Therefore, by using our new selected library (*n* = 1676) developed based on our in silico model, we would be able to reduce by 2.5-fold future in vitro primary screening efforts while increasing the hit rate. Further, most of the lead compounds (*n* = 17/18) and best hit compounds (*n* = 66/73) identified across our studies were accurately predicted by our in silico model (Supplementary File 1). However, it is important to note that the pre-selected SM library (*n* = 4182) is composed of a broad diversity of SM with different backbone structures and PCP profiles. Further, this study was based on the screens performed with bacterial pathogens grown in specific conditions (Table 1), which might not reflect the full antimicrobial potential of the pre-selected SM library, and therefore reducing the resolution of our prediction model. Performing additional screens against other plant, animal, and human pathogens using the same pre-selected library will further enhance the accuracy of our model and aid accelerated discovery of narrow- and broad-spectrum antimicrobials. In addition, it was observed that some PCP (Figure 1; e.g., geometrical shape coefficient, TPSA, and the number of oxygen) harbored a high contribution score among all the PCP studied, but these parameters did not display significant differences between antimicrobial activity profiles. By consequence, they may have contributed to some extent to the mis-prediction of our model.

Among the 60 PCP used in this study, MW, Zagreb group indexes, Kier shape, logP, MR, and the number of bonds (aromatic, heavy) had the highest predicted impact on the antimicrobial activity and spectrum of activity of the SM, which is in accordance with previous studies [31,32,33,34,35]. Interestingly, the precision of the in silico model was greater (up to 100% prediction accuracy) with broad-spectrum hits (SM with antimicrobial activity against at least seven species). Although only 7 SM were effective against at least seven species. A total of the 34 PCP were correlated with the activity spectrum of the SM. Several of these parameters (e.g., MR [32], logP [30,36], HBD [37], hydroxyl groups [38,39,40], aromatic groups [41], MW [31], carbons atoms [42], pyrophosphate [43], double bonds [44,45], and boron [46]) are known to influence the antimicrobial activity of a molecule. It was also shown that, for clusters X and W, the number of carboxylic acid groups and HBD per SM were positively correlated with the spectrum of activity, while the number of acyl groups per SM were negatively correlated. Similar observations were made with salvic acid derivatives in their antimicrobial properties when tested against *E. coli*, *Staphylococcus aureus*, and *Bacillus cereus* [37,47]. The in vitro analyses and docking studies revealed that the antimicrobial activity of salvic acid derivatives (labdanes and diterpenoids) was closely associated with the presence of the carboxylic acid groups in the molecules. The carboxylic group in the derivatives acted as HBD enhancing lipophilicity of the molecules, and thus modulated the interactions with the bacterial membrane [37,47]. It is important to notice that hydrogen bond acceptors (HBA) parameter was the only PCP strongly negatively correlated with the spectrum of activity of the SM, which coincides with the negative correlation observed between HBA and HBD (r^2^ = –0.47). 

## 4. Materials and Methods

### 4.1. Physico-Chemical Properties of the Pre-Selected SM Library and High Throughput Screening Data Associated with the Pre-Selected SM Library

Physico-chemical properties of the pre-selected library and the in vitro data from our earlier screening studies performed on plant, animal, and foodborne/human pathogens were used for in silico analyses in this study. A pre-selected library of 4182 bioactive SM obtained from ChemBridge (San Diego, CA, USA) was used in this study. Details about the prioritization methods used for the selection of the pre-selected library of SM is described in a previously published study [14]. A total of 60 PCP obtained using ChemBridge (hit2lead), PubChem, ChemMine, OpenBabel, and Joelib [48,49,50,51] were correlated with the antimicrobial activity of the SM (Table 2 and Supplemental File 4). The pre-selected SM library used for this study was deposited and is freely available in the following website: (http://chemogenomics.med.utoronto.ca/supplemental/bioactive/, accessed on 30 August 2021).

The in vitro data generated from our previously published drug discovery studies [15,16,17,18,19,20,21,22,23,24,25] using this SM library were used for in silico analyses. This library was previously screened against two foodborne (*Salmonella enterica* subsp. *Enterica* serotype Typhimurium JSG626 (ST) [16,18,23] and *Campylobacter jejuni* 81-176 (*Cj*) [17,22]), two animal (avian pathogenic *Escherichia coli* serotype O78 (APEC) [19,20] and *Mycoplasma gallisepticum* MG37 (Mg) [24]), and five plant pathogens (*Xanthomonas gardneri* SM761 (*Xg*) [25], *Xanthomonas perforans* SM775-12 (*Xp*) [25], *Acidovorax citrulli* Xu09-15 (*Ac*) [26], *Clavibacter michiganensis* subsp. *Michiganensis* C280 (*Cmm*) [15], and *Erwinia tracheiphila* TedCu10 (*Et*) [21]). The ST and APEC were also screened for virulence inhibitors [19,23] using this library and data associated with these screens were also included in the in silico analyses. Summary of the screening methodology and main findings from our previous studies are displayed in Table 1. Antimicrobial activity of the hits (SM that completely inhibited the growth (bacteriostatic or bactericidal effects) or attenuated at least 75% virulence of the bacterial pathogen at the designated concentration), SM screening conditions (growth medium, incubation conditions, small molecule concentration), taxonomy of the screened pathogens, hit rate (percentage of SM that completely inhibited the growth of the pathogen) were analyzed and organized from each study to determine the antimicrobial activity (hit versus non-hit) and spectrum of activity (number of pathogenic species inhibited) of the tested SM. For the in silico analyses, the in vitro data (antimicrobial activity and spectrum of activity of the SM) were used to correlate with the PCP of each compound to identify PCP profiles associated with the antimicrobial activity and spectrum of activity of the SM. A total of 2073 active SMs were identified across the nine pathogens screened from our previous studies (Supplemental File 1). Further, the activity spectrum of these 2073 compounds was determined by comparing the antimicrobial activity of the hits across all pathogens tested (Table 2 and Supplemental File 4). Veracity of our previously published in vitro data has been confirmed by resynthesizing all hit compounds identified during the primary screening and testing using similar screening conditions (Table 1). Details concerning the 2073 active SMs identified across the nine pathogens from our previous studies are presented in Supplemental File 1. Additional details concerning the antimicrobial activity and toxicity of SM in vitro and in vivo are available in our previously published studies [15,16,17,18,19,20,21,22,23,24,25]. 

### 4.2. Statistical Analyses

Only SM showing bacteriostatic or bactericidal activity (growth inhibitors) and the SM inhibiting the bacterial virulence (biofilm or quorum sensing inhibitors) at a given concentration and specific growing conditions were considered as “hit compounds” (confirmed antimicrobial activity; Table 1). A schematic of the methodology used in this study is described in Supplemental File 4. The analyses were performed using JMP PRO 14 software (SAS Institute, Cary, NC, USA). Three random subsets obtained by omitting 4.2–9.5% of the pre-selected library (*n* = 3785, 3819, and 4007 compounds per subset) were used to validate the prediction accuracy obtained using the in silico model presented in this study. Further, three random subsets of 10% of the SM molecule library were also used to validate the prediction accuracy of the model. Structural similarity of the SM was determined using Bartlett’s test and displayed using a hierarchical clustering method based on a Tanimoto score system (two-dimensional structure fingerprint with a single linkage algorithm). The contribution of each PCP on the antimicrobial (hits versus nonhits), spectrum (number of species a hit affected) of activity of the SM, and the type of inhibition (growth versus virulence inhibitors) was determined based on the bootstrap forest method. Statistical analyses were performed using one-way analysis of variance (ANOVA) for the antimicrobial activity and using simple linear regression for the spectrum of activity. The veracity of the significant discoveries was validated using a false discovery rate (FDR), equivalence test, and Huber M-estimation to identify only PCP large enough to be of pragmatic interest and reduce the impact of outliers on the statistical differences. A discriminant analyses was performed to predict the antimicrobial activity and spectrum of activity of the SM based on their PCP. A principal component analysis (PCA) was performed to identify clusterization patterns between SM based on their PCP. Similar analyses were performed to identify clustering patterns based on the pathogen type (plant, animal, and human pathogens). A Chi^2^ test combined with a Pearson and likelihood ratio test were used to identify hit rate differences between groups. A multivariate analysis was performed on the clusters generated by the PCA to identify correlation between the PCP and the antimicrobial activity and spectrum of activity of the SM. A scoring system was used to study the associations between the antimicrobial performance (spectrum of activity and hit rate) of the designated SM clusters and its PCP. The spectrum score was generated based on the number of hit compounds and the number of species affected by hit compounds (Supplemental File 3). The PCP score was generated based on the number of significant (*p* < 0.01) correlations between the spectrum of activity of the SM and their PCP (Figure 2C). Linear regression was used to assess the veracity of the in silico model (Figure 2D).

## 5. Conclusions

Overall, our study demonstrated that the antimicrobial activity and the spectrum of antimicrobial activity of SM were correlated with specific PCP (especially, MW, LogP, MR, Zagreb index, Kier shape, TPSA, MR, HBD, and HBA). These findings support the compound prioritization approach developed by Wallace et al. [14] (yeast-active screening; in silico prioritization using Lipinski rule; phenotype prediction using the naïve Bayes model), which allowed for building the original pre-selected library (*n* = 4.182 SM) used for this study. In addition, the in silico analyses performed in this study reduced the pre-selected library of 4182 compounds to 1676 narrow- or broad-spectrum compounds that truly possessed the antimicrobial activity and with predictable PCP. Therefore, virtual screening is a valuable tool to reduce the cost and time associated with drug discovery. Further, the screening data presented in this study would facilitate the development of novel derivatives likely to harbor a selective antimicrobial activity (narrow versus broad spectrum). In contrast, our analysis suggested the limited use of the two-dimension Tanimoto scoring system for the prediction of antimicrobial activity.

## 6. Patents

The following patents are associated with the lead compounds described in this study: US 62/608,335; US 62/697,876; US 16/083,811; and US 9,896,450.

## Figures and Tables

**Table 1:** Antimicrobial screening conditions and hit rates by species.

Bacterial Pathogens Screened	[SM] (μM)	Growing Conditions	Hit Rate (%)	References
**Growth Inhibitor Screenings**				
*Acidovorax citrulli* Xu9-15	100	50% NBY ^A^	1.4	Lu et al., 2020
*Clavibacter michiganensis* subsp *michiganensis* C280	100	NBY ^A^	11.2	Xu et al., 2016
*Erwinia tracheiphila* TedCu10	100	50% NBY ^A^	11.1	Vrisman et al., 2020
*Xanthomonas gardneri* SM761	100	MMX ^B^	29.3	Srivastava et al., 2020
*Xanthomonas perforans* SM755-12	100	MMX ^B^	17.9	Srivastava et al., 2020
Avian pathogenic *Escherichia coli* O78	100	M63 ^C^	1	Kathayat et al., 2019
*Mycoplasma gallisepticum* MG37	100	FREY ^C^	14.1	Helmy et al., 2020
*Campylobacter jejuni* 81–176	100	MH ^D^	18.7	Kumar et al., 2017
*Salmonella enterica* subsp. *enterica* serotype Typhimurium LT2	200	M9 ^C^	0.5	Deblais et al., 2018
**Virulence Inhibitor Screenings**				
Avian pathogenic *Escherichia coli* O78	100 ^A^	M63 ^C^	2.4 ^F^	Helmy et al., 2020
*Salmonella enterica* subsp. *enterica* serotype Typhimurium LT2	10 ^B^	TSB ^E^	5.2 ^G^	Koopman et al., 2015

[SM]: small molecule (SM) concentration used for screening; MMX: minimal medium for *Xanthomonas*; NBY: nutrient broth yeast extract; 50% NBY: one volume of NBY combined with one volume of sterile distilled water; MH: Mueller–Hinton; TSB: tryptic soy broth. ^A^ Aerobic, 24 h, at 28 °C; ^B^ Aerobic, 72 h, at 28 °C; ^C^ Aerobic, 12 h, at 37 °C; ^D^ Microaerophilic, 24 h, at 42 °C; ^E^ Aerobic, 24 h, at 30 °C. Hit: SM inhibiting the growth or virulence (^F^ quorum sensing or ^G^ biofilm) at a given SM concentration and growing condition.

**Table 2:** Antimicrobial and spectrum of activity of the SM by pathogen categories.

Antimicrobial Spectrum	Number of Hits per Pathogen Category			Number of Hits Across All the Pathogen Tested (*n* = 9)
	Plant (*n* = 5)	Animal (*n* = 2)	Foodborne (*n* = 2)	
Nonhit	2601	3491	3231	2109
1 species	752	651	885	926
2 species	438	40	66	442
3 species	243			277
4 species	135			202
5 species	13			135
6 species				59
7 species				23
8 species				8
9 species				1
Total number of hits across the whole pre-selected library (*n* = 4182)	1581	691	951	2073

Antimicrobial spectrum: number of species affected (growth or virulence inhibition) by a given SM (hit). The number of hits presented in columns 2–5 is independent between each column. Therefore, the sum of columns 2, 3, and 4 does not match the numbers in column 5 because some SM were effective against multiple pathogen categories (*n* = 819). Columns 2–4 display the number of hits effective against specific pathogen categories (plant (*n* = 5), animal (*n* = 2), or foodborne (*n* = 2) pathogens, respectively) and their associated activity spectrum (by rows). Column 5 displays the number of hits and their associated activity spectrum (by rows) across all three pathogen categories (*n* = 9). Empty cell: no data available.

**Table 3:** Lead compounds identified across different bacterial pathogens.

Screening Type	Bacterial Pathogens	Lead Compounds (Pubchem ID)																	
		1529361	2827372	1380897	2847561	2848076	5731123	16876368	42115615	42115777	45191821	45192477	25365835	45195011	42525758	25304876	25313118	42520454	45238750
GI	Et							X	X	X							X	X	
	Cmm												X		X				X
	Xp		X								X	X				X			
	Xg		X								X	X				X			
	APEC													X					
	Mg			X			X												
	Cj					X													
	ST			X	X														
VI	APEC	X																	

“X” means the lead compound significantly reduced the load of designated pathogens and severity of the disease in vivo with minimal impact on host, its microbiota, commensal bacteria, and probiotics/biocontrols. GI, growth inhibitors; VI, virulence inhibitors. Et, *Erwinia tracheiphila*; Cmm*, Clavibacter michiganensis michiganensis*; Xp, *Xanthomonas perforans*; Xg, *Xanthomonas gardneri*; APEC, avian pathogenic *E. coli*; Mg, *Mycoplasma gallisepticum*; Cj, *Campylobacter jejuni*; ST, *Salmonella enterica* subsp. *enterica* serotype Typhimurium.

**Table 4:** Impact of the physico-chemical properties of the pre-selected library on the antimicrobial activity of the small molecules.

Physico-Chemical Properties of the SM Used for This Study (*n* = 60)	Antimicrobial Activity (Hit versus Nonactive SM)		Spectrum of Activity (Nb of Species Affected by SM)		Growth Inhibitors versus Virulence Inhibitors		Lead Compounds versus Other Hits	
	Contribution Score	*p*-Value	Contribution Score	*p*-Value	Contribution Score	*p*-Value	Contribution Score	*p*-Value
Geometrical diameter	7.61	0.203	29.63	0.305	169.84	<0.001	6.25	0.253
Geometrical radius	0.07	0.021	0.46	<0.001	0.02	0.254	0.02	0.197
Kier shape 1	326.23	<0.001	568.69	<0.001	574.78	<0.001	6.76	0.739
Kier shape 2	99.72	<0.001	176.7	<0.001	138.93	<0.001	6.83	0.128
Lipophilicity (logP)	101.87	<0.001	134.83	<0.001	17.47	<0.001	12.21	0.071
Molar Refractivity	14,978.5	<0.001	26,375.9	<0.001	26,292.7	<0.001	572.9	0.426
Molecular weight	109,599	<0.001	166,671	<0.001	163,353	<0.001	1249.76	0.878
Number of acidic groups	0.04	0.423	0.53	0.001	0.02	0.757	0.18	0.009
Number of aromatic bonds	1617.33	<0.001	1952.13	<0.001	559.94	<0.001	13.63	0.632
Number of aromatic groups	37.09	<0.001	44.38	<0.001	22.15	<0.001	0.68	0.328
Number of atoms	1079.04	<0.001	2205.34	<0.001	4288.17	<0.001	187.23	0.177
Number of basic groups	3.08	0.004	8.42	0.001	5.5	<0.001	0.42	0.334
Number of bonds	1339.92	<0.001	2580.42	<0.001	4965.25	<0.001	252.18	0.101
Number of carbon	645.53	<0.001	959.85	<0.001	764.19	<0.001	29.41	0.120
Number of charges	0.31	0.031	1.57	0.002	0.27	0.066	2.31	<0.001
Number of chlorine	2.21	<0.001	3.85	<0.001	0.17	0.637	0.94	0.033
Number of double bonds	11.3	<0.001	18.39	<0.001	6.75	<0.001	4.49	0.010
Number of hydrogen	89.86	0.164	374.23	0.019	1185.4	<0.001	75.43	0.187
Number of halogens	5.22	0.004	16.37	<0.001	0.39	0.914	1.67	0.149
Number of HBA1	17.46	0.897	208.74	0.436	1140.71	<0.001	71.69	0.176
Number of HBA2	48.85	<0.001	93.98	<0.001	18.06	<0.001	3.71	0.123
Number of HBD 1	19.11	<0.001	47.64	<0.001	3.83	0.006	2	0.063
Number of HBD 2	10.67	<0.001	25.51	<0.001	2.05	0.051	0.58	0.550
Number of heavy bonds	800.79	<0.001	1195.37	<0.001	1320.5	<0.001	48.79	0.079
Number of heterocycles	0.51	0.613	5.26	0.048	19.56	<0.001	11.95	<0.001
Number of nitrogen	2.8	0.106	8.25	0.083	21.19	<0.001	0.47	0.697
Number of NO2	0.18	0.081	0.68	0.393	0.42	0.009	0.89	<0.001
Number of R-2NH	5.28	<0.001	19.71	<0.001	0.68	0.060	2.44	<0.001
Number of R-3N	6.55	<0.001	18.59	<0.001	1.25	0.117	3.3	0.006
Number of R-CN	0.01	0.349	0.03	0.194	0	0.839	0.12	<0.001
Number of R-COO-R	0.35	0.029	1.04	0.002	0.04	0.750	0.03	0.764
Number of R-COOH	0.03	0.072	0.25	<0.001	0.03	0.252	0.02	0.296
Number of R-OH	1.18	0.002	3.44	<0.001	1.36	0.138	1.54	0.087
Number of RINGS	14.73	<0.001	21.92	<0.001	22.23	<0.001	7.39	<0.001
Number of single bonds	48.57	0.991	625.48	0.501	2326.73	<0.001	269.52	0.111
Zagreb group index 1	27,153.5	<0.001	54,499.4	<0.001	133,000	<0.001	10,537.2	0.059
Zagreb group index 2	40,446.8	<0.001	71,160	<0.001	153,738	<0.001	11,507.2	0.042

Only PCP with significant differences are displayed in the table (*n* = 37/60). Orange cells indicate the selected PCP significantly contributed to the designated phenotype (*p* < 0.01 with a false discovery rate (FDR) LogWorth above 2). HBA: hydrogen bond acceptor; HBD: hydrogen bond donor. Nb: number. ND: not determined. The contribution of each PCP was determined based on the bootstrap forest method. Statistical analyses were performed using one-way ANOVA for the antimicrobial activity (hits versus non-hits) and using simple linear regression for the spectrum of activity (number of species a hit affected). Details about the PCP of the pre-selected library that were not associated with the antimicrobial activity of the SM are displayed in Supplemental File 2.

## Data Availability

The pre-selected SM library used for this study was deposited and is freely available in the following website: (http://chemogenomics.med.utoronto.ca/supplemental/bioactive/, accessed on 30 August 2021).

## Supplementary Materials

The following are available online at https://www.mdpi.com/article/10.3390/antibiotics10091065/s1. Supplemental File 1. Details concerning the 2073 hits identified across the eleven primary screenings; Supplemental File 2. Physico-chemical properties of the pre-selected library not associated with the antimicrobial activity of the small molecules; Supplemental File 3. Hit rate and spectrum of antimicrobial activity of the small molecules by cluster; Supplemental File 4. Virtual screening methodology used for the identification of narrow- and broad-spectrum small molecule antibacterials with predictable physico-chemical properties.

## Abbreviations

*Ac*: *Acidovorax citrulli*; ANOVA, analysis of variance, APEC, avian pathogenic *Escherichia coli*; *Cj*, *Campylobacter jejuni*; *Cmm*, *Clavibacter michiganensis* subsp. *michiganensis*; DMSO, dimethyl sulfoxide; *Et*, *Erwinia tracheiphila*; FDR, false discovery rate; Gp, group; HBA, hydrogen bound acceptors; HBD, hydrogen bound donors; LogP, lipophilicity; *Mg*, *Mycoplasma gallisepticum*; MR, molar refractivity; MW, molecular weight; Nb, number; ND, not determined; NS, nonsignificant; PCA, principal component analysis; PCP, physico-chemical property; SM, small molecule; ST, *Salmonella enterica* subsp. *enterica* serotype Typhimurium; TPSA, topological polar surface area; *Xg*, *Xanthomonas gardneri*; *Xp*, *Xanthomonas perforans*.

## References

[1] Reymond J.-L., Ruddigkeit L., Blum L., van Deursen R. (2012). The Enumeration of Chemical Space. WIREs Comput. Mol. Sci..

[2] Hughes J., Rees S., Kalindjian S., Philpott K. (2011). Principles of Early Drug Discovery. Br. J. Pharmacol..

[3] Simpkin V.L., Renwick M.J., Kelly R., Mossialos E. (2017). Incentivising Innovation in Antibiotic Drug Discovery and Development: Progress, Challenges and next Steps. J. Antibiot..

[4] Naylor N.R., Atun R., Zhu N., Kulasabanathan K., Silva S., Chatterjee A., Knight G.M., Robotham J.V. (2018). Estimating the Burden of Antimicrobial Resistance: A Systematic Literature Review. Antimicrob. Resist. Infect. Control.

[5] Shrestha P., Cooper B.S., Coast J., Oppong R., Do Thi Thuy N., Phodha T., Celhay O., Guerin P.J., Wertheim H., Lubell Y. (2018). Enumerating the Economic Cost of Antimicrobial Resistance per Antibiotic Consumed to Inform the Evaluation of Interventions Affecting Their Use. Antimicrob. Resist. Infect. Control..

[6] Segler M.H.S., Kogej T., Tyrchan C., Waller M.P. (2018). Generating Focused Molecule Libraries for Drug Discovery with Recurrent Neural Networks. ACS Cent. Sci..

[7] Rahman A., Choudhary M.I. (2017). Frontiers in Anti-Infective Drug Discovery.

[8] Ullah H., Ali S. (2017). Classification of Anti-Bacterial Agents and Their Functions. Antibact. Agents.

[9] Schneider G. (2013). Prediction of Drug-Like Properties.

[10] Bickerton G.R., Paolini G.V., Besnard J., Muresan S., Hopkins A.L. (2012). Quantifying the Chemical Beauty of Drugs. Nat. Chem..

[11] Vallianatou T., Giaginis C., Tsantili-Kakoulidou A. (2015). The Impact of Physicochemical and Molecular Properties in Drug Design: Navigation in the “Drug-like” Chemical Space. Adv. Exp. Med. Biol..

[12] Lipinski C.A., Lombardo F., Dominy B.W., Feeney P.J. (1997). Experimental and Computational Approaches to Estimate Solubility and Permeability in Drug Discovery and Development Settings. Adv. Drug Deliv. Rev..

[13] Mullard A. (2018). Re-Assessing the Rule of 5, Two Decades On. Nat. Rev. Drug Discov..

[14] Wallace I.M., Urbanus M.L., Luciani G.M., Burns A.R., Han M.K.L., Wang H., Arora K., Heisler L.E., Proctor M., Onge R.P.S. (2011). Compound Prioritization Methods Increase Rates of Chemical Probe Discovery in Model Organisms. Chem. Biol..

[15] Xu X., Kumar A., Deblais L., Pina-Mimbela R., Nislow C., Fuchs J.R., Miller S.A., Rajashekara G. (2015). Discovery of Novel Small Molecule Modulators of Clavibacter Michiganensis Subsp. Michiganensis. Front. Microbiol..

[16] Deblais L., Helmy Y.A., Kathayat D., Huang H., Miller S.A., Rajashekara G. (2018). Novel Imidazole and Methoxybenzylamine Growth Inhibitors Affecting Salmonella Cell Envelope Integrity and Its Persistence in Chickens. Sci. Rep..

[17] Deblais L., Helmy Y.A., Kumar A., Antwi J., Kathayat D., Acuna U.M., Huang H., de Blanco E.C., Fuchs J.R., Rajashekara G. (2019). Novel Narrow Spectrum Benzyl Thiophene Sulfonamide Derivatives to Control Campylobacter. J. Antibiot..

[18] Deblais L., Vrisman C., Kathayat D., Helmy Y.A., Miller S.A., Rajashekara G. (2019). Imidazole and Methoxybenzylamine Growth Inhibitors Reduce Salmonella Persistence in Tomato Plant Tissues. J. Food Prot..

[19] Helmy Y.A., Deblais L., Kassem I.I., Kathayat D., Rajashekara G. (2018). Novel Small Molecule Modulators of Quorum Sensing in Avian Pathogenic Escherichia Coli (APEC). Virulence.

[20] Kathayat D., Helmy Y.A., Deblais L., Rajashekara G. (2018). Novel Small Molecules Affecting Cell Membrane as Potential Therapeutics for Avian Pathogenic Escherichia Coli. Sci. Rep..

[21] Vrisman C.M., Deblais L., Helmy Y., Johnson R., Rajashekara G., Miller S.A. (2020). Discovery and Characterization of Low Molecular Weight Inhibitors of Erwinia Tracheiphila. Phytopathology.

[22] Kumar A., Drozd M., Pina-Mimbela R., Xu X., Helmy Y.A., Antwi J., Fuchs J.R., Nislow C., Templeton J., Blackall P.J. (2016). Novel Anti-Campylobacter Compounds Identified Using High Throughput Screening of a Pre-Selected Enriched Small Molecules Library. Front. Microbiol..

[23] Koopman J.A., Marshall J.M., Bhatiya A., Eguale T., Kwiek J.J., Gunn J.S. (2015). Inhibition of Salmonella Enterica Biofilm Formation Using Small-Molecule Adenosine Mimetics. Antimicrob. Agents Chemother..

[24] Helmy Y.A., Kathayat D., Ghanem M., Jung K., Closs G., Deblais L., Srivastava V., El-Gazzar M., Rajashekara G. (2020). Identification and Characterization of Novel Small Molecule Inhibitors to Control Mycoplasma Gallisepticum Infection in Chickens. Vet. Microbiol..

[25] Srivastava V., Deblais L., Kathayat D., Rotondo F., Helmy Y.A., Miller S.A., Rajashekara G. (2020). Novel Small Molecule Growth Inhibitors of Xanthomonas spp. Causing Bacterial Spot of Tomato. Phytopathology.

[26] Lu Y., Deblais L., Rajashekara G., Miller S.A., Helmy Y.A., Zhang H., Wu P., Qiu Y., Xu X. (2020). High-Throughput Screening Reveals Small Molecule Modulators Inhibitory to Acidovorax Citrulli. Plant Pathol..

[27] Iredell J., Brown J., Tagg K. (2016). Antibiotic Resistance in Enterobacteriaceae: Mechanisms and Clinical Implications. BMJ.

[28] Partridge S.R. (2015). Resistance Mechanisms in Enterobacteriaceae. Pathology.

[29] O’Shea R., Moser H.E. (2008). Physicochemical Properties of Antibacterial Compounds: Implications for Drug Discovery. J. Med. Chem..

[30] Ebejer J.-P., Charlton M.H., Finn P.W. (2016). Are the Physicochemical Properties of Antibacterial Compounds Really Different from Other Drugs?. J. Cheminformatics.

[31] Sahariah P., Cibor D., Zielińska D., Hjálmarsdóttir M.Á., Stawski D., Másson M. (2019). The Effect of Molecular Weight on the Antibacterial Activity of N,N,N-Trimethyl Chitosan (TMC). Int. J. Mol. Sci..

[32] Cho C.-W., Park J.-S., Stolte S., Yun Y.-S. (2016). Modelling for Antimicrobial Activities of Ionic Liquids towards Escherichia Coli, Staphylococcus Aureus and Candida Albicans Using Linear Free Energy Relationship Descriptors. J. Hazard. Mater..

[33] Mugumbate G., Overington J.P. (2015). The Relationship between Target-Class and the Physicochemical Properties of Antibacterial Drugs. Bioorganic Med. Chem..

[34] Bajaj S., Sambi S.S., Madan A.K. (2005). Prediction of Anti-Inflammatory Activity of N-Arylanthranilic Acids: Computational Approach Using Refined Zagreb Indices. Croat. Chem. Acta.

[35] Verma V., Singh K., Kumar D., Narasimhan B. (2017). QSAR Studies of Antimicrobial Activity of 1,3-Disubstituted-1H-Naphtho[1,2-e][1,3]Oxazines Using Topological Descriptors. Arab. J. Chem..

[36] Rezaee S., Khalaj A., Adibpour N., Saffary M. (2009). Correlation between Lipophilicity and Antimicrobial Activity of Some 2-(4-Substituted Phenyl)-3(2H)-Isothiazolones. Daru-J. Fac. Pharm..

[37] Echeverría J., Urzúa A., Sanhueza L., Wilkens M. (2017). Enhanced Antibacterial Activity of Ent-Labdane Derivatives of Salvic Acid (7α-Hydroxy-8(17)-Ent-Labden-15-Oic Acid): Effect of Lipophilicity and the Hydrogen Bonding Role in Bacterial Membrane Interaction. Molecules.

[38] Chibane L.B., Degraeve P., Ferhout H., Bouajila J., Oulahal N. (2019). Plant Antimicrobial Polyphenols as Potential Natural Food Preservatives. J. Sci. Food Agric..

[39] Arfa A.B., Combes S., Preziosi-Belloy L., Gontard N., Chalier P. (2006). Antimicrobial Activity of Carvacrol Related to Its Chemical Structure. Lett. Appl. Microbiol..

[40] Echeverría J., Opazo J., Mendoza L., Urzúa A., Wilkens M. (2017). Structure-Activity and Lipophilicity Relationships of Selected Antibacterial Natural Flavones and Flavanones of Chilean Flora. Molecules.

[41] Swamy M.K., Akhtar M.S., Sinniah U.R. Antimicrobial Properties of Plant Essential Oils against Human Pathogens and Their Mode of Action: An Updated Review. https://www.hindawi.com/journals/ecam/2016/3012462/.

[42] Almeida R.P.P., Killiny N., Newman K.L., Chatterjee S., Ionescu M., Lindow S.E. (2012). Contribution of RpfB to Cell-to-Cell Signal Synthesis, Virulence, and Vector Transmission of Xylella Fastidiosa. Mol. Plant Microbe Interact..

[43] ’t Hart P., Wood T.M., Tehrani K.H.M.E., van Harten R.M., Śleszyńska M., Rentero Rebollo I., Hendrickx A.P.A., Willems R.J.L., Breukink E., Martin N.I. (2017). De Novo Identification of Lipid II Binding Lipopeptides with Antibacterial Activity against Vancomycin-Resistant Bacteria. Chem. Sci..

[44] Hsiao C.P., Siebert K.J. (1999). Modeling the Inhibitory Effects of Organic Acids on Bacteria. Int. J. Food Microbiol..

[45] Totaro G., Cruciani L., Vannini M., Mazzola G., Di Gioia D., Celli A., Sisti L. (2014). Synthesis of Castor Oil-Derived Polyesters with Antimicrobial Activity. Eur. Polym. J..

[46] Sayin Z., Ucan U.S., Sakmanoglu A. (2016). Antibacterial and Antibiofilm Effects of Boron on Different Bacteria. Biol. Trace Elem. Res..

[47] Urzúa A., Rezende M.C., Mascayano C., Vásquez L. (2008). A Structure-Activity Study of Antibacterial Diterpenoids. Molecules.

[48] Backman T.W.H., Cao Y., Girke T. (2011). ChemMine Tools: An Online Service for Analyzing and Clustering Small Molecules. Nucleic Acids Res..

[49] Kim S., Chen J., Cheng T., Gindulyte A., He J., He S., Li Q., Shoemaker B.A., Thiessen P.A., Yu B. (2019). PubChem 2019 Update: Improved Access to Chemical Data. Nucleic Acids Res..

[50] O’Boyle N.M., Banck M., James C.A., Morley C., Vandermeersch T., Hutchison G.R. (2011). Open Babel: An Open Chemical Toolbox. J. Cheminformatics.

[51] Guha R., Howard M.T., Hutchison G.R., Murray-Rust P., Rzepa H., Steinbeck C., Wegner J., Willighagen E.L. (2006). The Blue Obelisk—Interoperability in Chemical Informatics. J. Chem. Inf. Model..

