# Supplementary material for: Compound Prioritization through Meta-Analysis Enhances the Discovery of Antimicrobial Hits against Bacterial Pathogens
Source: Antibiotics (Basel). 2021 Sep 2;10(9):1065. doi: 10.3390/antibiotics10091065 (PMC8471430; doi:10.3390/antibiotics10091065)

Supplemental File 2. Physico-chemical properties of the pre-selected library not associated with the antimicrobial activity of the small molecules

Physico-chemical properties of the SM used for this study (n=23)	**Antimicrobial activity** (hit versus non-active SM)		**Spectrum of activity** (Nb of species affected by SM)		**Growth inhibitors versus Virulence inhibitors**		**Lead compounds versus other hits**	
	**Contribution score**	**P-value**	**Contribution score**	**P-value**	**Contribution score**	**P-value**	**Contribution score**	**P-value**
**Fraction of rotatable bonds**	0.03	0.51405	0.00	0.52604	0.02537	0.00178	0.0106	0.05658
**Geometrical shape coefficient**	4628.00	0.30204	383.83	0.69106	56.7194	0.8863	584.486	0.11072
**Number of aliphatic OH groups**	1.36	0.02906	0.09	0.48943	0.09586	0.47312	0.62397	0.01465
**Number of B atoms**	0.00	0.5785	0.00	0.3132	2.5E-05	0.83039	7.9E-05	0.6862
**Number of Br atoms**	0.58	0.07177	0.01	0.93614	0.20987	0.00128	0.09635	0.04494
**Number of F atoms**	7.60	0.08808	1.04	0.21977	0.29172	0.77832	0.41109	0.82956
**Number of hydrophobic groups**	0.00		0.00		0		0	
**Number of I atoms**	0.03	0.37273	0.00	0.29034	0.00071	0.61683	0.00119	0.91064
**Number of N atoms**	8.71	0.08316	3.22	0.10603	0.63704	0.6969		
**Number of nF**	6.98	0.08808	1.02	0.21977	0.3669	0.77832	0.28421	0.82956
**Number of O atoms**	11.91	0.02065	2.41	0.13297	1.28054	0.55295	0.97759	0.55188
**Number of OSO groups**	0.00		0.00		0		0	
**Number of P atoms**	0.01	0.69733	0.00	0.64077	0.00016	0.7105	0.00047	0.48386
**Number of RCCH**	0.23	0.16492	0.08	0.04798	0.04006	0.20856	0.08337	0.07515
**Number of RCHO**	0.00	0.31062	0.00	0.16086	0		0	
**Number of RCOR**	1.37	0.59653	0.22	0.14906	0.06454	0.72424	0.06874	0.61002
**Number of ROPO3**	0.00	0.23332	0.00	0.3132	1.9E-05	0.83039	7.5E-05	0.6862
**Number of ROR**	5.22	0.98708	1.03	0.229	1.53539	0.08666	1.36187	0.13758
**Number of S atoms**	1.61	0.0816	0.12	0.80021	0.10859	0.57068	0.20031	0.31616
**Number of SO groups**	0.00		0.00		0		0	
**Number of SO2 groups**	0.39	0.05126	0.15	0.08393	0.24094	0.02401	0.28689	0.0126
**Number of triple bonds**	0.08	0.62568	0.02	0.37567	0.0634	0.22733	0.03132	0.23175
**Topical polar surface area (TPSA)**	3720.57	0.06982	389.40	0.43542	956.779	0.12008	334.467	0.42421

Supplemental File 3. Hit rate and spectrum of antimicrobial activity of the small molecules by cluster

Clusters	Spectrum of activity (species affected by a given SM; in %)									Spectrum score
	0	1	2	3	4	5	6	7	8	
Cluster V (n=594)	65	22	6.7	2.7	3.4	0	0	0	0	57.3
Cluster W (n=1,396)	53	18	16	7.2	3.7	1.9	0.4	0.1	0	98.8
Cluster X (n=404)	64	16	9.6	5	3.5	1.8	0.3	0	0	74.6
Cluster Y (n=1,355)	48	16	17	9	6.2	3.1	1	0.1	0	123.2
Cluster Z (n=433)	33	17	22	14	8.8	3.7	1.6	0	0.2	168.1

The color of the cells is proportional to their value (percentage; the number of small molecules [SM] with the designated spectrum of antimicrobial activity compared to the whole population of the selected cluster; a total of 9 species tested for each compound). The spectrum score was generated by multiplying the number of hit compounds (in %) by the number of species affected by hit compounds.

Supplemental File 4. Virtual screening methodology used for the identification of narrow- and broad- spectrum small molecule anti-bacterials with predictable physico-chemical properties

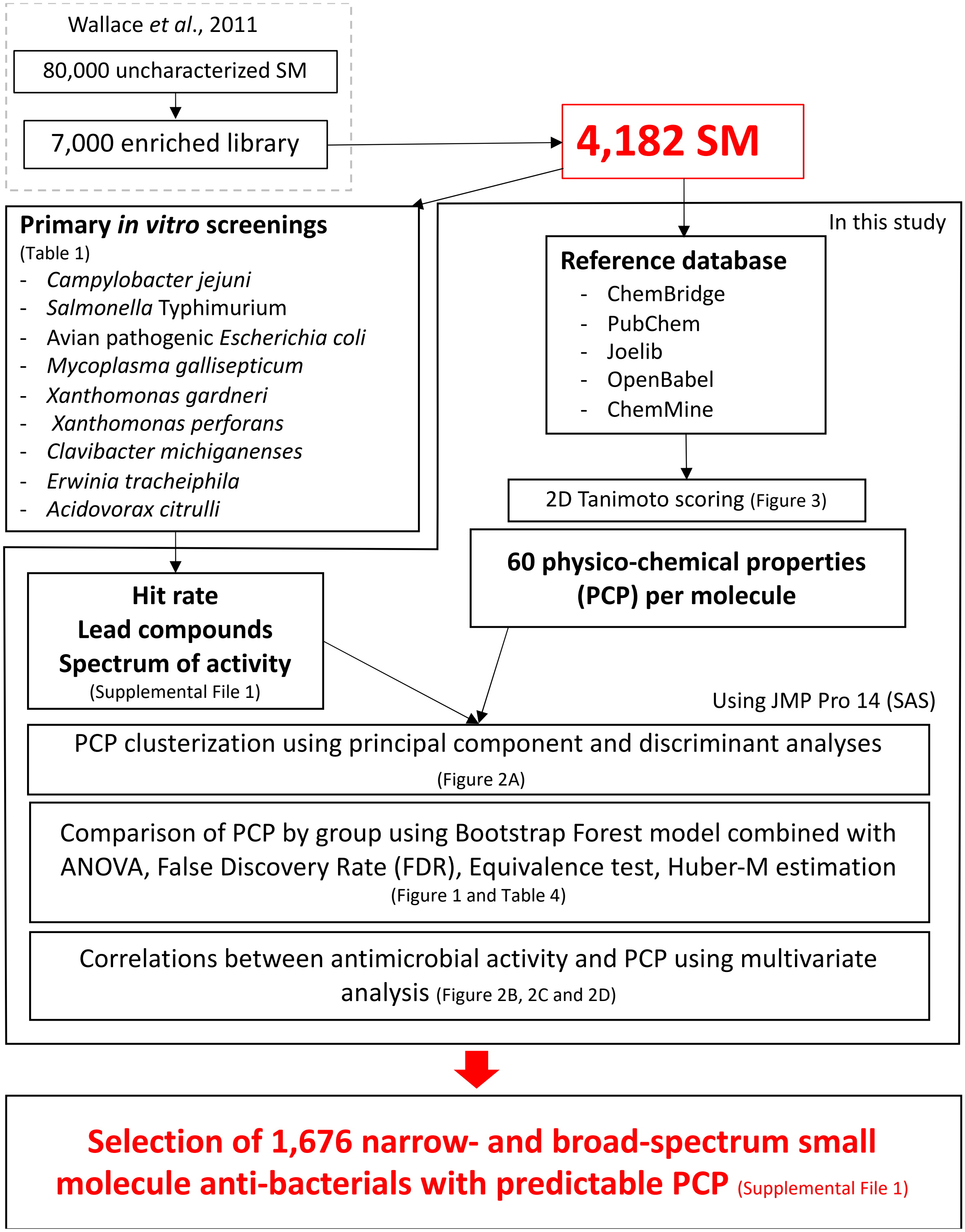

Supplement: Supplementary file 1 [file antibiotics-10-01065-s001.zip › Supplemental_Files 2-4_8-28-21.pdf]
